# Patient-provider communication about cognition and the role of memory concerns: a descriptive study

**DOI:** 10.1186/s12877-023-04053-3

**Published:** 2023-05-31

**Authors:** Nikki L. Hill, Emily Bratlee-Whitaker, Heejung Jang, Sakshi Bhargava, Andrea Yevchak Sillner, Justin Do, Jacqueline Mogle

**Affiliations:** 1 Ross and Carol Nese College of Nursing, Penn State University, 201 Nursing Sciences Building, University Park, PA 16802 USA; 2 RTI Health Solutions, 3040 East Cornwallis Road, PO Box 12194, Research Triangle Park, NC 27709 USA; 3 Department of Psychology, Clemson University, 418 Brackett Hall, Clemson, SC 29634 USA; 4 Sidney Kimmel Medical College, 1025 Walnut St, Philadelphia, PA 19107 USA

**Keywords:** Patient-provider communication, Subjective cognition, Help-seeking, Cognition, Memory complaints

## Abstract

**Background:**

Early identification of cognitive impairment is an important part of health promotion in aging. However, many older adults do not seek help for cognitive problems until their ability to function independently is substantially impacted. The purpose of this descriptive study was to explore older adults’ experiences with patient-provider communication specific to cognition as well as compare barriers and facilitators between those with and without memory concerns.

**Methods:**

We conducted an online survey with individuals aged 65 + years (n = 409; mean age = 71.4(4.73); 54% female; 79% non-Hispanic White), purposively sampled to include those with and without memory concerns. Questionnaires included measures of subjective memory decline (SMD), memory concerns, past healthcare experiences, as well as open-ended questions regarding patient-provider communication about cognition. Content analysis was used to code open-ended responses. Logistic regression was used to examine differences in facilitators and barriers to communication among three groups: no SMD (n = 130), SMD without memory concerns (n = 143), and SMD with memory concerns (n = 136).

**Results:**

Only 16.6% of participants reported discussing cognition with a healthcare provider. Of the remaining 83.4%, approximately two-thirds would be open to such discussions in certain circumstances, most frequently if they had worsening memory problems. Over half of participants reported that their provider had never offered cognitive testing. Compared to the no SMD and SMD without memory concerns groups, participants reporting SMD with memory concerns were more likely to: (1) discuss cognition if their healthcare provider initiated the conversation, and (2) avoid discussions of cognitive problems due to fears of losing independence.

**Conclusions:**

We found that most participants, including those reporting SMD with memory concerns, had never discussed cognition with their healthcare providers. Patient-reported barriers and facilitators to communication about cognition differed in several areas based on SMD status and the presence or absence of memory concerns. Consideration of these differences can guide future efforts to improve early identification of subtle cognitive changes that would benefit from further monitoring or intervention.

## Background

Maximizing cognitive health in aging is a national priority well-aligned with individual goals. In a meta-synthesis of qualitative evidence, van Leeuwen and colleagues identified preservation of cognitive health as key quality of life element in 41 of 48 studies reviewed [1]. As evidence builds regarding early intervention targeting modifiable health and lifestyle factors to support cognitive health (e.g., hypertension, social connectedness), the importance of early detection of cognitive changes is consistently supported [2, 3]. Indeed, it is estimated that 40% of Alzheimer’s disease (AD) cases could be prevented or delayed with early intervention [4]. However, early detection of cognitive impairment is a continued challenge: up to 90% of mild cognitive impairment (MCI) cases go undetected in the U.S. [5], and less than 20% of older adults with cognitive concerns discuss them with their healthcare provider [6]. Identification of early cognitive changes, such as MCI, as well as implementation of interventions supporting cognitive health, depend on patient-provider communication, yet few studies have examined older adults’ perspectives on such pivotal conversations [7].

Several national efforts encourage cognitive screening in primary care as a tool to improve early communication about cognitive health. Most notably, the Centers for Medicare and Medicaid Services recommend cognitive screening as a component of the Medicare Annual Wellness Visit [8]. All U.S. adults aged 65 or older who have Medicare coverage are eligible for this yearly visit, and multiple organizations such as the Alzheimer’s Association have highlighted its potential for improving the detection of cognitive impairment in primary care [9]. However, routine cognitive screening for all older adults is not universally recommended due to insufficient evidence regarding the benefits and limitations [10]. In addition to the lack of consensus regarding screening, healthcare providers are limited in scope and time allocated to see patients in primary and community-based settings. Unless an older patient presents with a cognitive complaint, assessment may focus on other aspects of health, such as management of chronic conditions. Therefore, detecting the first signs of cognitive impairment relies largely on the patient, their family members, or other care partners to actively share cognitive issues or concerns with a healthcare provider.

Subjective memory decline (SMD) is characterized by the perception of a decline in memory ability when objective cognitive testing indicates normal performance [11], and is often most noticeable during everyday tasks when cognitive demands are high [12]. SMD can indicate a prodromal stage of AD or other dementia, prior to MCI. Indeed, older adults with SMD, compared to those without, are up to four times more likely to develop MCI or dementia [13]. However, not all older adults with SMD go on to develop more substantial impairments. Recent work has attempted to increase the specificity and sensitivity of SMD as a predictor of future dementia risk through the inclusion of whether the individual is worried about these changes [14, 15]. SMD with memory concerns specifically, rather than SMD alone, is related to higher risk for MCI development over a one-year period [16] as well as elevated levels of biomarkers thought related to AD [17]. Such evidence suggests an important role for SMD, as well as worry about these perceived changes, in identifying individuals at greater risk for future cognitive decline.

It is estimated that the overall rate of cognitive impairment among adults 65 and older living in the community is about 25%, with higher rates of impairment and dementia with increasing age [6, 18]. Similarly, SMD prevalence tends to increase with age overall [19]. However, across the adult life span, SMD has been found to gradually increase until about age 55, decrease during the next decade, and then substantially increase around age 75 [20]. Therefore, better understanding of patient-provider communication about cognition in those 65 and older is particularly important since cognitive impairment risk is higher, and perceptions of memory decline are known to change during this later life period.

Several systematic reviews have identified barriers and facilitators to seeking help for cognitive impairment or dementia, concluding that older adults consider a variety of factors such as the severity of problems, potential benefits or consequences of disclosing concerns, as well as past interactions with healthcare providers [7, 21–23]. However, older adults and their care partners typically do not seek help until cognitive problems are more severe and everyday functional abilities are impacted [21, 23]. Therefore, there is a need to better understand communication between older patients and healthcare providers about cognition prior to the onset of cognitive impairment that impairs daily function (i.e., before problems are viewed as severe enough to necessitate seeking a diagnosis). Past studies have retrospectively examined factors influencing help-seeking among older adults with SMD [24, 25], MCI [26], and dementia [27]; however, evidence regarding patients’ discussions with providers about cognition outside of the process of seeking help for cognitive concerns is largely limited to patient reports of whether cognitive screening was offered [6]. Furthermore, prior research has not examined the role of SMD and memory concerns in such discussions: it is not known whether patient-provider communication about cognition differs between those with and without SMD, and further, whether concerns about perceived decline in memory may play a role in barriers or facilitators of these discussions.

The purpose of this descriptive study was to: (1) explore older adults’ experiences with and perspectives on patient-provider communication specific to cognition, and (2) compare barriers and facilitators to discussions of cognition among older adults with and without SMD, including whether they are concerned about a perceived decline in memory performance. To our knowledge, this is the first study to report patient-reported factors that may influence future discussions with their healthcare provider about cognition as well as to examine differences based on SMD with and without memory concerns.

## Methods

### Procedures & sample

The current investigation was part of a larger cross-sectional study examining perceptions of aging and cognition in community-dwelling older adults. An online survey was conducted with individuals aged 65 years or older in the United States (n = 409), purposively sampled to represent diversity in demographic characteristics as well as those with and without memory concerns. Specifically, we aimed to enroll a maximum of 80% of participants who were non-Hispanic White, approximately 25% with less than a high school education, no more than 50% with education beyond high school, and an approximate 50/50 split between female and male participants. We further purposively sampled based on memory concerns with approximately one-third of participants in each of the following categories: no SMD, SMD without memory concerns, SMD with memory concerns. All participants met the following inclusion criteria: able to speak and read English, no self-reported diagnosis of dementia or other cognitive impairment, and live independently (i.e., not a resident of an assisted living or skilled care nursing facility).

Participants were recruited through Qualtrics Online Panels [28]. Qualtrics maintains a database of individuals who have opted in to receive invitations to participate in studies for which they meet eligibility criteria. Participants receive compensation directly through the Qualtrics platform and are informed of compensation details prior to providing informed consent. This study was approved by the (University blinded for review) Institutional Review Board.

The full survey took approximately 30 min to complete; portions of the collected data are included in the current study. Both quantitative and qualitative (i.e., open-ended) data were collected. Multiple methods were used to ensure quality responses including an internal quality review of all completed surveys and the inclusion of five attention check questions throughout the survey. For each survey section, open-ended questions were asked first to avoid response bias due to related questionnaires.

### Measures

**Demographics and Health Status.** Participants responded to questions about their age, gender, marital status, race/ethnicity, income, education, and residential area. In addition, participants rated their current health on a 5-point scale (1 = Excellent; 5 = Poor) and their health now compared to one year ago (1 = Much better; 5 = Much worse) [29].

**Subjective Memory Decline (SMD).** SMD was assessed with a single question used in previous research examining early symptoms of cognitive decline [30, 31]: “Do you feel like your memory is becoming worse?” Participants responded on a 3-point scale (1 = no; 2 = yes, but this does not worry me; 3 = yes, this worries me) that supports examination of self-perceptions of memory decline as well as associated concerns.

**Patient-Provider Communication about Cognition.** At the beginning of the survey, “cognition” was defined for participants as: “your ability to think, concentrate, and remember.” To explore patient-provider communication about cognition we first asked: “Have you ever talked to a doctor about your cognition?” Participants responded on a dichotomous scale (1 = yes; 2 = no). Those who responded “Yes” were then asked who they talked to (1 = primary care provider; 2 = neurologist; 3 = memory clinic specialist; 4 = psychiatrist/psychologist; 5 = other) as well as an open-ended question: “Please tell us about your conversation,” with the additional probes, “What prompted you to have a discussion,” “What did you talk about,” and, “What was the outcome?”). Those who responded “No,” they had never talked to a doctor about their cognition, were asked “Would you ever discuss your cognition with a healthcare provider?” with the additional probe, “Why or why not?”

To further understand whether participants had conversations about cognition with their primary healthcare providers specifically, all participants were then asked the following question: “Which of the following best describes experiences with your primary healthcare provider? Please check all that apply.” See Table 4 for checklist of response options.

**Table 1:** Descriptive Characteristics of the Sample

Characteristics	Total (*n* = 409)	No SMD (*n* = 130)	SMD without memory concerns (*n* = 143)	SMD with memory concerns (*n* = 136)
Age in years, *M* (SD)	71.42 (4.73)	71.99 (5.01)	71.71 (4.47)	70.56 (4.63)
Gender, *n* (%)				
Male	187 (45.72)	54 (41.54)	71 (49.65)	62 (45.59)
Female	221 (54.03)	75 (57.69)	72 (50.35)	74 (54.41)
Other – open-ended	1 (0.24)	1 (0.77)	--	--
Race/Ethnicity, *n* (%)				
Non-Hispanic White	323 (78.97)	97 (74.62)	115 (80.42)	111 (81.62)
Black	44 (10.76)	23 (17.69)	11 (7.69)	10 (7.35)
Hispanic/Latino	18 (4.40)	6 (4.62)	4 (2.80)	8 (5.88)
American Indian/ Alaska Native	2 (0.49)	--	1 (0.70)	1 (0.74)
Asian	17 (4.16)	3 (2.31)	10 (6.99)	4 (2.94)
Not listed – open-ended	5 (1.22)	1 (0.77)	2 (1.40)	2 (1.47)
Annual Family Income, *n* (%)				
< $10,000	5 (1.25)	2 (1.61)	2 (1.41)	1 (0.74)
$10,001 - $20,000	54 (13.47)	20 (16.13)	13 (9.15)	21 (15.56)
$20,001 - $40,000	121 (30.17)	45 (36.29)	44 (30.99)	32 (23.70)
$40,001 - $60,000	83 (20.70)	23 (18.55)	31 (21.83)	29 (21.48)
$60,001 - $80,000	63 (15.71)	18 (14.52)	25 (17.61)	20 (14.81)
$80,001 - $100,000	26 (6.48)	6 (4.84)	7 (4.93)	13 (9.63)
> $100,000	49 (12.22)	10 (8.06)	20 (14.08)	19 (14.07)
Highest degree completed, *n* (%)				
Less than high school degree	12 (2.93)	7 (5.38)	4 (2.80)	1 (0.74)
High school degree or GED	93 (22.74)	32 (24.62)	32 (22.38)	29 (21.32)
Vocation or technical degree	17 (4.16)	5 (3.85)	5 (3.50)	7 (5.15)
Some college but no degree	113 (27.63)	39 (30.00)	40 (27.97)	34 (25.00)
Associate’s degree	28 (6.85)	3 (2.31)	11 (7.69)	14 (10.29)
Bachelor’s degree	89 (21.76)	23 (17.69)	33 (23.08)	33 (24.26)
Graduate degree	57 (13.94)	21 (16.15)	18 (12.59)	18 (13.24)
Residential Area, *n* (%)				
Large city	71 (17.36)	24 (18.46)	23 (16.08)	24 (17.65)
Suburb near a large city	172 (42.05)	51 (39.23)	64 (44.76)	57 (41.91)
Small city or town	86 (21.03)	30 (23.08)	29 (20.28)	27 (19.85)
Rural area	80 (19.56)	25 (19.23)	27 (18.88)	28 (20.59)

**Facilitators and Barriers to Patient-Provider Communication.** Two checklists were used to measure [1] facilitators and [2] barriers to patient communication with healthcare providers about cognition. Checklists were adapted from a measure published by Pearman [32] and extended to include additional items based on our previous systematic review of older adults’ help-seeking for cognitive problems [7]. Participants were asked, “Which of the following would make you more likely to talk to a healthcare provider about your memory, thinking, or concentration?” and “Do any of the following make you less likely to talk to a healthcare provider about your memory, thinking, or concentration?”. Participants could select multiple responses from a list of 14 and 11 options, respectively. See Tables 5 and 6 for all response options.

**Table 2:** Participant Perspectives on Future Patient-Provider Discussions about Cognition (n = 341)

Response Codes	Sample Frequency	Exemplar Quotes
**Yes or Maybe, Would Discuss Cognition with Provider (n = 250, 73.3%) Why or Why Not?**		
If experiencing cognitive problems	26.0%	*I would if I were having problems but at this time I’m not so I would not*
If a healthcare provider could help (e.g., possible treatments)	18.0%	*Yes I would talk to my provider about my cognition if needed, as I believe that it perhaps could be something that could be addressed or corrected when detected early enough to prevent more serious problems later*
If cognitive problems were getting worse	15.6%	*I would if I thought it was getting really bad. At the moment after to talking to my family and friends I don’t seem to be any worse off than any of them*
If cognitive problems were affecting daily life	8.4%	*I would if I ever thought that it would interfere with my day to day living. Such as forget to turn off a stove burner or something basic like how to dress myself*
Not sure/undecided	8.0%	*I’m not sure i have never thought about it*
If a family member/friend expressed concern	7.2%	*When I am to the point to where others are telling me that something is wrong when I am around them then I will begin to have an interest in talking to my healthcare provider about what is happening*
To get answers or a diagnosis (e.g., cognitive testing)	7.2%	*I believe that it is normal to see a decline in cognitive skills as we age. It could also mean that there may be concern that correlates with testing for Alzheimer’s disease or dementia*
To be proactive about cognitive health	5.6%	*Yes, because it is part of my overall well-being*
Trust in healthcare provider	5.2%	*Sure because I trust my doctor and would not be afraid to talk to him about this. He might could help me*
**No, Would Not Discuss Cognition with Provider (n = 91, 26.7%) Why or Why Not?**		
Not experiencing cognitive problems	47.3%	*Haven’t and will not as I don’t see any problem areas*
Cognitive problems experienced aren’t serious enough	20.9%	*I think the change is very minor, so I do not think it needs to be discussed. There is no short-term memory loss*
Cognitive problems experienced are normal aging	18.7%	*I really don’t feel that I should be concerned. The issues that I have I feel like they are just a regular decline that most people go through with aging*
Embarrassment or fear about discussing cognitive problems	9.9%	*I would never discuss any problems with my memory or thinking with a healthcare provider. Both of my parents had dementia in their 90s, and I do not want to discuss that with anyone. Nope, not gonna do that*
Lack of trust or value in healthcare providers	8.8%	*I haven’t seen a doctor in years and have no intention of seeing one. I am not interested in discussing my health with any sort of medical people*
Healthcare provider does not discuss it at visits	5.5%	*They have never asked any question regarding this issue*

**Table 3:** Participant Experiences with Patient-Provider Discussions about Cognition (n = 68)

Response Codes	Sample Frequency	Exemplar Quotes
Sought input on specific cognitive concerns	38.2%	*We discussed in general terms the fact that I am having more difficulty recalling words, since I have very random conversations. Most days I do little more than exchange pleasantries to people in passing. When I was actively teaching, coaching, etc., words flowed more easily. Many times, this searching for words or common names is disconcerting*
Provider normalized/dismissed cognitive concerns	32.4%	*In the beginning I just expressed concern over forgetfulness. Discussed if medication could cause this. It was pretty much dismissed and didn’t get feel that there was concern. More recently, I have noted more impairment and it worries me. But my primary care physician doesn’t really offer any comment or alternative*
Discussion of cognitive concerns led to assessment	25.0%	*My mother died of Alzheimer’s as did other members of her family so I was concerned and talked to my PCP about some minor memory issues. He did a cognitive test and said that I was fine for my age*
Cognition discussion was related to other health issues	20.6%	*I have had several mental health diagnoses since I was a teenager and I also have chronic pain and was misdiagnosed with a stroke last year*
Discussion or assessment as part of routine exam	11.8%	*I turned 65 and got Medicare. So I went for a wellness appointment as I hadn’t been in the habit to see doctors unless injured. I did what I think was a test for dementia, or to check my cognitive skills. I passed, but talked about forgetfulness possibly due to not concentrating at the moment about where I set something down or moves something*
Provider decided to continue to monitor for change	8.8%	*We discussed how things were going and I told her about forgetting things more. Simple things. We discussed what things and she said there were tests she could conduct but did not feel like I had reached a point to worry and that we would check back later. Which we did on each visit after*
Sought input on normal cognitive changes in aging	5.9%	*I just asked my doctor if it was normal for a person my age to have memory loss*
Provider recommended non-pharmacological interventions for cognition	5.9%	*Initially I was aware of forgetting then remembering words while speaking to friends, but most of them also had the same issue… The doctor suggested I play games, do crossword puzzles, etc. to help with the issue*
Sought information on prevention of cognitive decline	5.9%	*Because my mom went through this problem I wanted to know was I going to have this problem and how could I look at it now before it starts to affect me*
Referral to specialist	5.9%	*I spoke about it with my psychologist who recommended that I have a neuropsychological battery of tests done… The psychologist said it was rather like running state-of-the-art software on a windows 98 operating system. All the components were there and running as they should, just slower*

### Data analysis

Descriptive statistics were calculated in IBM SPSS Statistics 29 for all quantitative measures and are provided in Table 1. Differences in facilitators and barriers by SMD group (i.e., No SMD, SMD without memory concerns, SMD with memory concerns) were examined using logistic regression in SAS 9.4 to determine odds ratios (OR) and 95% confidence intervals (CI) of pairwise differences between each SMD group. Statistical significance was calculated using a significance level of 0.05, and sample frequency of agreement calculated for each facilitator and barrier.

**Table 4:** Experiences with Primary Care Providers (n = 409)

Response Options	Frequency
My healthcare provider has never offered cognitive testing.	60.9%
I have not discussed cognitive/brain health with my healthcare provider because there’s been no need.	54.1%
My healthcare provider has never brought up the topic of cognitive/brain health.	41.6%
My healthcare provider offers cognitive testing at my annual wellness visit or physical.	23.1%
My healthcare provider gives me guidance on cognitive/brain health.	14.3%
I participate in the cognitive testing offered by my healthcare provider at my visits.	13.1%
My healthcare provider answers my questions about cognitive/brain health.	12.9%
My healthcare provider explains the purpose of cognitive testing to me.	8.4%
My healthcare provider explains my cognitive testing results to me.	8.4%
I have not felt comfortable discussing cognitive/brain health with my healthcare provider.	4.1%
I completed a cognitive test with my healthcare provider in the past but chose not to do one again.	2.0%
I decline cognitive testing offered by my healthcare provider at my visits.	0.5%

Content analysis [33] was used to code and categorize open-ended survey responses in Excel. Beginning with open coding, two team members experienced in qualitative analysis independently read responses line-by-line and developed inductive codes based on words, phrases, and statements in participant responses. Subsequently, these codes were discussed, compared, and revised until a consensus was reached among all team members on a comprehensive codebook. Finally, each code was compared and contrasted to identify higher level categories descriptive of participant responses.

## Results

### Participant characteristics

Table 1 includes demographic and self-reported health status information for the sample, including representation across SMD groups. The average age was 71.4 (SD = 4.73); most participants were female (n = 221, 54.0%) and non-Hispanic White (n = 323, 79.0%). The proportion of the sample in each SMD group was as follows: 31.8% no SMD (n = 130), 35.0% SMD without memory concerns, and 33.3% SMD with memory concerns (n = 136).

**Experiences with patient-provider communication about cognition.** Most participants (n = 341, 83.4%) reported having never discussed cognition with a healthcare provider. Of these, when asked whether they “would ever” discuss cognition with a healthcare provider, 66.9% said they would, 26.7% said they would not, and 6.5% said they might. The reasons for these answers, their frequencies, and exemplar quotes are provided in Table 2, based on the coded open-ended responses. The most common reasons participants would discuss cognition with a provider in the future were: if they were experiencing cognitive problems (26.0%), if they thought their healthcare provider could help with cognitive problems (e.g., possible treatments, 18.0%), and if their cognitive problems were getting worse (15.6%). The most common reasons participants would not discuss cognition with a provider in the future were: they do not have cognitive problems (47.3%), their cognitive problems aren’t serious enough (20.9%), and the cognitive problems they experience are perceived to be a normal part of aging (18.7%).

**Table 5:** Facilitators of Patient-Provider Communication about Cognition by SMD Group

Facilitator Endorsement	Frequency (%)				OR (95% CI)		
**Which of the following would make you more likely to talk to a healthcare provider about your memory, thinking, or concentration?**	**Overall**	**Group 1**: **No SMD**	**Group 2**: **SMD without memory concerns**	**Group 3**: **SMD with memory concerns**	**Group** **1 vs. 2**	**Group** **1 vs. 3**	**Group** **2 vs. 3**
If memory or thinking problems were interfering with my life	280 (68.46)	86 (66.15)	98 (68.53)	96 (70.59)	0.90 (0.54–1.49)	0.81 (0.49–1.37)	0.91 (0.55–1.51)
If my memory or thinking problems were getting worse	247 (60.39)	65 (50.00)	94 (65.73)	88 (64.71)	**0.52 ** **(0.32–0.85)**	**0.55** **(0.33–0.89)**	1.05 (0.64–1.71)
If I started having new memory or thinking problems	239 (58.44)	71 (54.62)	85 (59.44)	83 (61.03)	0.82 (0.51–1.33)	0.77 (0.47–1.25)	0.94 (0.58–1.51)
If I felt worried or concerned about memory or thinking problems	235 (57.46)	70 (53.85)	86 (60.14)	79 (58.09)	0.77 (0.48–1.25)	0.84 (0.52–1.37)	1.09 (0.68–1.76)
If my friends or family thought I should talk to my healthcare provider	185 (45.23)	49 (37.69)	76 (53.15)	60 (44.12)	**0.53** **(0.33–0.87)**	0.77 (0.47–1.25)	1.44 (0.90–2.30)
If my healthcare provider asked me about my memory or thinking abilities	171 (41.81)	40 (30.77)	58 (40.56)	73 (53.68)	0.65 (0.40–1.07)	**0.38** **(0.23–0.63)**	**0.59** **(0.37–0.95)**
Trusting my healthcare provider	116 (28.36)	32 (24.62)	40 (27.97)	44 (32.35)	0.84 (0.49–1.44)	0.68 (0.40–1.17)	0.81 (0.49–1.36)
If sharing these experiences would help my long-term health (for example, give my healthcare provider more information to guide their recommendations)	105 (25.67)	22 (16.92)	42 (29.37)	41 (30.15)	**0.49** **(0.27–0.88)**	**0.47** **(0.26–0.85)**	0.96 (0.58–1.61)
Having good communication with my healthcare provider	102 (24.94)	29 (22.31)	29 (20.28)	44 (32.25)	1.13 (0.63–2.02)	0.60 (0.35–1.04)	**0.53** **(0.31–0.92)**
If I knew that others in my family had dementia or Alzheimer’s disease	89 (21.76)	22 (16.92)	30 (20.98)	37 (27.21)	0.77 (0.42–1.41)	**0.55** **(0.30–0.99)**	0.71 (0.41–1.23)
If I heard of a new drug or non-drug treatment that might be able to help me	45 (11.00)	11 (8.46)	11 (7.69)	23 (16.91)	1.11 (0.46–2.65)	**0.45** **(0.21–0.97)**	**0.41** **(0.19–0.88)**
If my friends or family came with me to my healthcare provider visit/appointment	28 (6.85)	9 (6.92)	10 (6.99)	9 (6.62)	0.99 (0.39–2.52)	1.05 (0.40–2.73)	1.06 (0.42–2.70)
Nothing - I would not talk to my health provider about memory or thinking problems	9 (2.20)	8 (6.15)	1 (0.70)	0 (0.00)	--	--	--

*Note.* SMD = Subjective Memory Decline. OR = Odds Ratio. CI = Confidence Interval. OR estimates in bold indicate significant group differences at p < .05

Of the 16.6% of the sample (n = 68) who reported discussing cognition with a healthcare provider in the past, the specific provider(s) they talked to were: 86.8% primary care provider, 19.1% psychiatrist/psychologist, 16.2% neurologist, and 4.4% memory clinic specialist. Most commonly, participants described seeking their provider’s advice for specific cognitive concerns (38.2%), normalization or dismissal of concerns by their provider (32.4%) and completing cognitive testing following reporting of concerns to their provider (25.0%). Additional participant descriptions, their frequencies, and exemplar quotes are provided in Table 3, based on the coded open-ended responses.

**Table 6:** Barriers to Patient-Provider Communication about Cognition by SMD Group

Barrier Endorsement	Frequency (%)				OR (95% CI)		
**Do any of the following make you less likely to talk to a healthcare provider about your memory, thinking, or concentration?**	**Overall**	**Group 1**: **No SMD**	**Group 2**: **SMD without memory concerns**	**Group 3**: **SMD with memory concerns**	**Group** **1 vs. 2**	**Group** **1 vs. 3**	**Group** **2 vs. 3**
Nothing - I would always talk to my health provider about memory or thinking problems	250 (61.12)	99 (76.15)	83 (58.04)	68 (50.00)	**2.31** **(1.37–3.89)**	**3.19** **(1.89–5.40)**	1.38 (0.86–2.22)
I believe memory changes are normal for people my age	129 (31.54)	12 (9.23)	52 (36.36)	65 (47.79)	**0.18** **(0.09–0.35)**	**0.11** **(0.06–0.22)**	0.62 (0.39–1.01)
I am afraid of losing my independence	55 (13.45)	9 (6.92)	18 (12.59)	28 (20.59)	0.52 (0.22–1.19)	**0.29** **(0.13–0.64)**	0.56 (0.29–1.06)
I think there is nothing anyone can do to help memory or thinking problems	26 (6.36)	4 (3.08)	3 (2.10)	19 (13.97)	1.48 (0.33–6.75)	**0.20** **(0.07–0.59)**	**0.13** **(0.04–0.46)**
If my friends or family were pushing me to ask for help	26 (6.36)	9 (6.92)	12 (8.39)	5 (3.68)	0.81 (0.33–2.00)	1.95 (0.64–5.98)	2.40 (0.82–7.00)
I would not want anybody to know I was having memory or thinking problems	25 (6.11)	4 (3.08)	6 (4.20)	15 (11.03)	0.73 (0.20–2.63)	**0.26** **(0.08–0.79)**	**0.35** **(0.13–0.94)**
I am afraid I won’t be taken seriously	16 (3.91)	3 (2.31)	3 (2.10)	10 (7.35)	1.10 (0.22–5.56)	0.30 (0.08–1.11)	0.27 (0.07–1.00)
I believe that my memory is my own business and not anybody else’s	12 (2.93)	4 (3.08)	2 (1.40)	6 (4.41)	2.24 (0.40–12.42)	0.69 (0.19–2.50)	0.31 (0.06–1.55)
I do not want to know if I have cognitive impairment	12 (2.93)	2 (1.54)	1 (0.70)	9 (6.62)	2.22 (0.20–24.76)	0.22 (0.05–1.04)	**0.10** **(0.01–0.80)**
I don’t have access to a healthcare provider (for example, lack of transportation or insurance coverage)	3 (0.73)	3 (2.31)	0 (0.00)	0 (0.00)	--	--	--

*Note.* SMD = Subjective Memory Decline. OR = Odds Ratio. CI = Confidence Interval. OR estimates in bold indicate significant group differences at p < .05

All participants (n = 409) were asked to respond to the checklist of items describing past experiences with their primary healthcare provider, specific to cognition. The most common responses related to a lack of communication about cognition in primary care were: “My healthcare provider has never offered cognitive testing” (60.9%), “I have not discussed cognitive/brain health with my healthcare provider because there’s been no need” (54.1%), and “My healthcare provider has never brought up the topic of cognitive/brain health” (41.6%). See Table 4 for frequencies of all response options.

**Facilitators of Patient-Provider Communication about Cognition by SMD group.** Table 5 presents frequencies of facilitators of patient-provider communication about cognition across SMD groups. The most common facilitators aligned with the severity of cognitive problems (e.g., “If memory or thinking problems were interfering with my life”). Both SMD groups (with and without memory concerns) were significantly more likely than the non-SMD group to report they would seek help from a healthcare provider if they experienced worsening memory and thinking problems (OR = 0.55 (95% CI 0.33-0.89); OR = 0.52 (95% CI: 0.32-0.85), respectively). Social support was another common facilitator. Compared to the non-SMD group (OR = 0.53; 95% CI: 0.33-0.87), the SMD without memory concerns group was more likely to say they would seek help if friends or family thought they should discuss cognitive concerns with a healthcare provider.

Healthcare providers also played an important role in whether older adults would discuss cognitive concerns. Relative to those in the SMD without memory concerns and the non-SMD groups, members of the SMD with memory concerns group were more likely to discuss their concerns if their healthcare provider initiated the conversation (OR = 0.59 (95% CI: 0.37-0.95); OR = 0.38 (95% CI: 0.23-0.63), respectively). Further, the frequency of sharing memory concerns if they would support long-term health was significantly higher among the SMD groups (with and without memory concerns) than in the non-SMD group (OR = 0.47 (95% CI: 0.26-0.85); OR = 0.49 (95% CI: 0.27-0.88), respectively). Finally, relative to the SMD without memory concerns group, the SMD with memory concerns group was more likely to say they would bring up their concerns if they had good communication with their healthcare provider (OR = 0.53 (95% CI: 0.31-0.92)).

Having a family history of dementia or AD was another common facilitator of communication, with a difference identified between the SMD with memory concerns group and the non-SMD group (OR = 0.55; 95% CI: 0.30-0.99). Further, the likelihood of communicating with a healthcare provider if they heard about a new treatment that might be able to help was significantly higher among the SMD with memory concerns group than among both the non-SMD group and the SMD without memory concerns group (OR = 0.45 (95% CI: 0.21-0.97); OR = 0.41 (95% CI: 0.19-0.88)).

**Barriers to Patient-Provider Communication about Cognition by SMD group.** Table 6 presents frequencies of barriers to patient-provider communication about cognition across SMD groups. The most frequent response from participants (n = 250) was that nothing would prevent them from communicating with providers about cognitive concerns. This response was more common among the non-SMD group than among the SMD with and without memory concerns groups (OR = 3.19 (95% CI: 1.89–5.40); OR = 2.31 (95% CI: 1.37–3.89), respectively). The next most common barriers reported were the normalization of problems (n = 129), fear of losing independence (n = 55), and a lack of benefits to having such discussions (n = 26). Specifically, the non-SMD group was less likely than the SMD with and without memory concerns groups to believe memory changes are normal for people their age (OR = 0.11 (95% CI: 0.06-0.22); OR = 0.18 (95% CI: 0.09-0.35), respectively). In addition, relative to the non-SMD group, the SMD with memory concerns group reported being more likely to avoid communication about cognitive problems because they were afraid of losing their independence and because they thought there is nothing anyone can do to help memory or thinking problems (OR = 0.29; 95% CI: 0.13-0.64; OR = 0.20, respectively).

Further, the SMD with memory concerns group was significantly more likely to report they would not want anyone to know they were having memory or thinking problems than the non-SMD and SMD without memory concerns groups (OR = 0.26 (95% CI: 0.08-0.79); OR = 0.35 (95% CI: 0.13-0.94), respectively). A somewhat similar pattern emerged regarding participants’ own knowledge of potential problems— the SMD with memory concerns group was significantly more likely to report that they would not want to know if they had cognitive impairment than the SMD without memory concerns group (OR = 0.10 (95% CI: 0.01-0.80)). In this case, however, the difference between the SMD with memory concerns group and the non-SMD group was not statistically significant.

## Discussion

This descriptive study explored older adults’ experiences with and intentions for communicating with healthcare providers about cognition, identified factors that facilitate and hinder such conversations from the patient perspective, and examined how these factors vary by memory concerns. Specifically, we investigated differences by SMD status in combination with the presence or absence of concerns about a perceived decline in memory, given the higher risk for cognitive decline among older adults who report SMD with related concerns [14]. Overall, we found that over 80% of participants had not discussed cognition with a healthcare provider, although they also reported they would in certain circumstances (e.g., worsening cognitive problems). Facilitators and barriers to patient-provider communication about cognition differed by SMD status in several areas; consideration of these can guide future efforts to improve early identification of subtle cognitive changes that would benefit from further monitoring or intervention.

Despite the inclusion of cognitive screening as a component of the Medicare Annual Wellness Visit since 2011 [9], most participants reported that their primary healthcare provider had never offered cognitive testing. Furthermore, over 40% of participants reported that their primary healthcare provider has never brought up the topic of cognitive or brain health. Our findings align with previous research highlighting that only 16% of older adults report completing cognitive testing at primary care visits [6]. Clinical recommendations guide providers on testing procedures, but this is primarily initiated when patients or family members disclose cognitive concerns [9]. Our findings extend understanding of factors that contribute to older patients’ decisions to initiate discussions about cognition with their healthcare providers. Specifically, among participants who had never discussed cognition with a provider, over two-thirds indicated they would in certain circumstances including if they experienced new or worsening cognitive problems and if cognitive problems were affecting their daily lives. In addition, over 40% of participants reported they would be likely to talk to their healthcare provider about cognition if their provider asked them. These findings highlight how current practices do not necessarily align with national initiatives to identify cognitive impairment at its earliest stages, before everyday function and well-being is impacted. As Werner [34] noted, older adults may only be willing to seek help when their problems are severe enough to interfere with daily function.

Previous research has examined older adults’ experiences seeking help for cognitive problems, including the process of MCI and dementia diagnosis [21, 23]. However, our findings extend this understanding to factors that influence broader conversations about cognition and cognitive change in aging, as well as older adults’ perspectives on what may influence such conversations in the future. Furthermore, the use of open-ended questions and coding of qualitative data along with checklists derived from previous literature [7, 32] provides, to our knowledge, the first comprehensive description of older adult-reported facilitators and barriers to communication with providers about cognition. Overall, patient facilitators were related to the intensity (e.g., getting worse) or impact (e.g., interfering with life) of cognitive problems, relationships with healthcare providers (e.g., patient-provider trust), and social support. Older adults who seek help from their providers for cognitive problems tend to be experiencing more troubling sequalae compared to non-help-seekers, such as withdrawal from typical activities [35]. On average, patients wait up to four years after troubling symptoms begin to discuss them with a healthcare provider, citing embarrassment and worry as common reasons for the delay [36]. This orientation to communication about cognition is problematic for multiple reasons as brain health is part of overall health, and intervening for maximum benefit is time-sensitive: the earlier the better [4]. As Begum et al. [24] found, help-seekers may view their healthcare providers as helpful and perceive discussion of memory concerns as similar to discussions of other health-related needs, while non-help-seekers may think they will not be taken seriously or will be wasting their provider’s time. Our results add further support to the importance of the patient-provider relationship in creating an environment where older adults are comfortable and confident in discussing memory concerns before they progress to the point of substantially impacting function and well-being.

Due to the association between SMD and future risk for cognitive decline [37, 38], older adults with SMD may be a prime group to target for cognitive impairment detection efforts. However, the heterogeneity of SMD is a known limitation of previous research. Evidence suggests that concerns about perceived memory decline, rather than SMD alone, are a better predictor of MCI and dementia risk, possibly because concerns indicate a larger functional impact and more substantial decline [12]. Our findings suggest that memory concerns may also be an important consideration in approaches to improve patient-provider communication about cognition, an important addition to past research on help-seeking in SMD. We found that, compared to the non-SMD and SMD without memory concerns groups, the SMD with memory concerns group would be more likely discuss cognition with their healthcare provider when the provider initiated the conversation. Therefore, perceived memory decline alone seems to be less of a factor in such conversations than concern about the decline specifically. Previous research suggests that providers initiate such discussions when they notice potential problems during a routine visit and pursue further assessment [39]. However, healthcare providers will not know how to best support patients’ cognitive health if they are not aware of whether the patient is experiencing new or increasing cognitive problems. Further consideration of how to support patients in initiating conversations is critical. Cognitive assessment is not a one-sided process; patient participation is critical to its initiation and execution [40].

The most common barrier to patient-provider communication identified was the normalization of cognitive problems, including older adults viewing the problems they experience as part of the normal aging process. This view was more common among participants who had SMD than those who did not. Previous research has shown that older adults who do not have a family history of dementia are more likely to attribute memory problems to a consequence of normal aging [41]. Family history may heighten sensitivity to cognitive problems due to personal experiences or patients perceiving they have a biomedical risk factor due to heredity [7]. The role of familial risk in help-seeking for memory concerns was identified in a previous small qualitative study [24]. Our findings confirm the importance of this facilitating factor in a large sample with diverse demographic characteristics as well as those with and without concerns about their experience of SMD. Further, a lack of knowledge about cognitive health may increase the avoidance of help-seeking behaviors [41]. These patterns may indicate that when older adults recognize true signs of cognitive problems, they are willing to seek help from professionals, but when they do not recognize signs, they tolerate these symptoms and accept them as a normal part of aging for a longer time.

Our findings also indicate that relative to those in other groups, those who had SMD with memory concerns were more fearful of losing their independence and more likely to not want anyone to know they are having memory problems. Importantly, prior research has found that stigmatized beliefs about dementia prevent discussion about cognitive problems within informal networks [42]. Some older adults believe that seeking help for cognitive problems and potentially learning about a diagnosis will not have any benefit; thus, extant stigma, shame, embarrassment, and fear threaten their well-being [34]. This algins with findings of systematic reviews of physician-reported barriers to cognitive screening in primary care: healthcare providers fail to offer screenings when they perceive patient reluctance or concerns about the stigma of a diagnosis [43, 44]. The reduction of stigma and the empowerment of persons with memory concerns and their families is a promising area for improvement, with a focus on public awareness and knowledge about living well with cognitive impairment. For example, the dementia-friendly communities created by the World Health Organization and Alzheimer’s Disease International implement strategies to ensure that patients with dementia, their families, and their informal networks feel confident that when they seek help, they will be supported and understood rather than risk losing their identity or being stigmatized and isolated [21]. There is a need to extend similar initiatives to support individuals earlier in the trajectory of cognitive decline.

Additionally, there were several differences between the SMD with and without memory concerns groups. Compared to those with without memory concerns, older adults with concerns were less likely to want to know if they have cognitive impairment and were also more likely to rely on their provider to initiate the conversation. While SMD with memory concerns can indicate the beginning of cognitive decline [15, 16], it may also be related to participants’ fear of public stigma and/or their own stereotypes about cognitive problems (self-stigma), both of which reduce patients’ willingness to seek an assessment [9, 34]. Internalized or self-stigma may be especially likely to deter individuals from seeking treatment and social services, even when opportunities are available, in the interest of avoiding the stigma associated with certain diagnostic labels [45]. As Wills and DePaulo [46] suggested, individuals may choose not to seek professional help as a way to protect themselves from embarrassment and feelings of inferiority or incompetence. Further, because stigma tends to spread from the stigmatized individuals to their close connections, family members may experience stigma, increased emotional distress, and social isolation [45].

This study did have several limitations that are important to consider and build upon in future work. Although we were purposive in our sampling strategy to obtain diversity in demographic characteristics as well as those with and without SMD and memory concerns, most of our sample identified as White and non-Hispanic. Greater representation across race and ethnicity is needed in future studies for more generalizable results. Relatedly, we did not use random sampling; individuals who volunteered to participate in this study may have different experiences or beliefs about their cognition than those who did not choose to participate. Furthermore, the online survey format of the study may have contributed to sampling bias as well as limited our ability to screen for cognitive impairment since we relied on participant self-report of diagnoses. In addition, we used open-ended online survey questions to obtain qualitative data, which did not allow for probing for additional detail that could have expanded and informed our results. However, online surveys as a qualitative research tool offer unique strengths, including providing a more anonymous venue for participants to share sensitive beliefs [47], such as beliefs about cognition and interactions with healthcare providers.

Our investigation of patient-provider communication about cognition focused solely on older adults’ perspectives of their healthcare interactions. Future research should consider both patient and provider perspectives, as well as consider examining the concordance or discordance of their experiences; identifying miscommunication or misunderstanding during discussions about cognition may provide opportunities for intervention development. In addition, we examined differences in patient-provider communication based on SMD group and memory concerns, not other aspects of subjective cognition or cognitive concerns. In their review of subjective cognition measures used in research across eight countries, the Subjective Cognitive Decline Initiative Working Group found that memory was the most common cognitive domain assessed [48]. However, their recommendations for research on subjective cognition included differentiating reports of cognitive decline with and without associated concerns (as implemented in the current study) as well as expanding assessment of other cognitive domains and related non-cognitive measures (e.g., mood, personality). Future work on patient-provider communication should also consider these factors to further our understanding of barriers and facilitators.

## Conclusion

Earlier identification of AD and its frequent precursor, MCI [49], provides a critical window of opportunity for evidence-based interventions to promote functional independence, psychosocial health, potentially delay or slow cognitive decline [4, 50–52], as well as for patients to build a care team, access services, enroll in clinical trials, and plan for the future [53]. Despite national support for improving detection of cognitive impairment in older adults [9], up to 90% of MCI cases are not identified by a healthcare provider [5]. We found that older adults’ communication with their health providers about cognition remains limited, as does the use of cognitive screening at routine healthcare visits. Individuals with SMD, and particularly those with associated memory concerns, have a higher risk for future cognitive decline and AD compared to their peers without SMD [13]. Our findings suggest that although this group may have a higher need for interventions to promote cognitive and functional health, they reported being more fearful of losing their independence and of others knowing about their cognitive problems, as well as relying on the healthcare providers to initiate the discussion. Therefore, future efforts to improve early detection of cognitive impairment should consider tailoring interventions to address these specific barriers to patient-provider communication about cognition.

## Data Availability

The dataset generated and analyzed during the current study is not publicly available in compliance with the consent for data use provided by study participants but is available from the corresponding author on reasonable request.
